# Differing Professional Perspectives on the Interprofessional Collaboration in IPUs: A Mixed-methods Study

**DOI:** 10.5334/ijic.7516

**Published:** 2023-08-10

**Authors:** Dorine J. van Staalduinen, Petra E. A. van den Bekerom, Sandra M. Groeneveld, Arie Franx, Anne M. Stiggelbout, M. Elske van den Akker-van Marle

**Affiliations:** 1 Medical Decision Making, Department of Biomedical Data Sciences, Leiden University Medical Center, Albinusdreef 2, 2333 ZA, Leiden, The Netherlands; 2 Institute of Public Administration, Leiden University, Turfmarkt 99, 2511 DP, Den Haag, The Netherlands; 3 Erasmus University Medical Center, Doctor Molewaterplein 40, 3015 GD, Rotterdam, The Netherlands

**Keywords:** value-based healthcare, Integrated Practice Unit, integration of care, interprofessional collaboration, teamwork, team membership, professional interaction

## Abstract

**Introduction:**

An important aspect of Value-Based Healthcare (VBHC) is providing the full cycle of care for a specific medical condition through interprofessional collaboration. This requires employees from diverse professional backgrounds to interact, but there is limited knowledge on how professionals perceive such interprofessional collaboration. We aimed to provide insight into how different professionals perceive Integrated Practice Unit (IPU) composition and what factors influence the quality of interprofessional collaboration within IPUs.

**Methods:**

A survey was administered to employees from different professional backgrounds (medical specialists, nurses, allied health professionals, administrative employees) working in IPUs to assess their perception of the composition of their IPU and the quality of the interactions. Subsequently, semi-structured interviews were conducted to gain a deeper understanding of the findings of the survey.

**Results:**

Medical specialists and nurses were most frequently considered to be part of an IPU and indicated that they have high quality interactions. Allied health professionals were less often considered part of the team by all other professional groups and all report low quality interaction with this group. The extent to which a professional group is perceived as a team member depends on their visibility, involvement in the treatment of the patient, and shared interest. Differences in the quality of interprofessional collaboration are influenced by organizational structures, knowledge of each other’s expertise, and by ways of communication.

**Conclusions:**

In VBHC, there seems to be a lack of common perception of an IPU’s composition and a failure to always achieve high quality interprofessional collaboration. Given the importance of interprofessional collaboration in VBHC, effort should be invested in achieving a shared understanding and improved collaboration.

## 1. Introduction

Value-based healthcare (VBHC) is a management practice that focuses on value-driven rather than volume-driven healthcare, and it is receiving growing attention in healthcare organisations [1,2]. A key element of VBHC is the provision of the full cycle of care for a specific medical condition in an Integrated Practice Unit (IPU) that consists of professionals with different functional backgrounds (e.g. medical specialists, nurses, allied health professionals and administrative employees) who then work together as a team [1,3,4,5]. Establishing IPUs consisting of team members representing different professional groups has been proposed on the premise that this would enhance the quality of the delivered care in comparison with relatively more monodisciplinary healthcare teams [5,6].

Adequately shaping a team’s composition is seen as one of the challenges in optimizing collaboration and comfort within such IPUs [7]. Previous VBHC research shows that organising the delivery of care in IPUs with professionals from different professional groups is challenging, in part due to confusion between professional groups regarding interests, tasks, and responsibilities [1].

Interprofessional collaboration enables members with diverse functional backgrounds to exchange clinical knowledge and skills for the optimal treatment of a patient [8]. This requires recognition of the roles of the different professionals in a team, as well as a willingness to understand the work of other professions [9,10]. Interprofessional teams are associated with increased team effectiveness, increased empowerment, greater job satisfaction and reduced employee turnover [11,12]. However, in order to achieve these positive outcomes, interaction among the team members is crucial [13].

Differing perspectives among the healthcare professionals regarding what constitutes effective collaboration can make collaboration complicated and challenging. Earlier research on interprofessional collaboration in healthcare teams has indicated that perceptions of team membership, team effectiveness and high-quality collaboration may vary between disciplines [14,15,16,17,18]. For example, Reeves and Lewin (2004) found that doctors view interprofessional collaboration as involving medical, rather than non-medical, colleagues [16]. Others argue that perceptions regarding collaboration between professional groups may differ by professional group, possibly due to different professional norms and values in their work activities [19]. For instance, collaboration between doctors and nurses has been found to be more business-like, whereas collaboration between nurses and other staff was characterised as friendly and less rushed [16]. Similar results regarding differences in collaboration style between doctors, nurses and other staff members have been found in non-hospital healthcare settings [20,21].

Although interprofessional collaboration is at the heart of VBHC, and essential if it is to be effective, there is a lack of studies investigating interprofessional collaboration in IPUs [2]. We therefore set out to answer the following research question: How do IPU members from different professional backgrounds perceive the composition of the IPU and the quality of interprofessional collaboration within it?

## 2. Methods

### 2.1. Study design and setting

This study used a mixed-method design in which qualitative data from semi-structured interviews were used to explain findings based on quantitative data collected through a survey. A mixed-method design was chosen to achieve an in-depth understanding of the results. The study was conducted in two Dutch academic hospitals in 2021 and 2022. Both hospitals had started to implement IPUs as part of the hospital-wide VBHC strategy around 2017. Earlier research has described IPUs as theoretically an ideal type of organizational unit within VBHC theory that requires major organizational change [5,22]. The IPUs in the two hospitals were similar and were mainly focused on improving the quality of care through increasing coordination and measuring patient-related outcome measures (PROMs). These IPUs delivered, for instance, oncology or chronic care. The IPUs in this study are similar to the IPUs described in VBHC theory, except they do not have financial accountability.

The independent medical ethics committees of the two hospitals agreed that the study did not fall within the Medical Research Act and thereby that ethical approval was not needed. Informed consent was ensured through written and oral communication with all participants who were further told that they were able to withdraw from the study at any time.

### 2.2. Sampling

Prior to data collection, a list was made of members of the various professional groups that were formally involved in IPUs. This list was established in collaboration with project managers responsible for the VBHC implementation. Professionals involved in an IPU who worked as a medical specialist, nurse, administrative employee, or allied health professional were all eligible to participate in the survey. In this study, allied health professionals are those who provide health-related services separate from medicine and nursing, for instance, physiotherapists and dieticians. The identified eligible professionals (N = 235) received the online survey between January and December 2021. The survey ended with a question asking participants if they would be willing to take part in an interview. Based on the answers to this question, professionals from different disciplinary backgrounds were invited for an interview. To include a wide range of perspectives, participants were purposively sampled.

### 2.3. Data collection

#### 2.3.1. Survey

To evaluate the perceived IPU composition and the quality of interprofessional collaboration in the IPUs, team perception and Relational Coordination were measured. Perception, in this study, refers to the way in which professionals interpret their IPU and the collaboration with others in the IPU. The VBHC literature argues that IPUs should consist of several professional groups: “who devote a significant amount of their time to the medical condition and see themselves as part of a common organizational unit” [5]. Investigating who is considered part of the IPU by the different professional groups gives a first insight into how IPUs are perceived. Team perception was operationalized as perceived team size and team diversity, similarly to Doekhie et al. (2017), by including the following questions: “How many members does your IPU consist of?” and “Which of the following professional groups do you consider part of your IPU (medical specialist, nurse, allied health professional, administrative employee)?” [15].

Relational Coordination (RC) is a concept used to assess the quality of communication and relationships among members of interprofessional teams [23]. The RC literature views good quality collaboration as existing in relationships that have 1) shared goals, 2) shared knowledge and 3) mutual respect; plus communication that is 4) frequent, 5) timely, 6) accurate and 7) problem-solving. High levels of RC indicate effective coordination across professional boundaries. In this study, RC was measured using the Dutch translation of Gittell’s RC Survey [24] that involves Likert-scale responses from 1.00 (never) to 5.00 (always) to statements on each of the seven RC elements.

#### 2.3.2. Interviews

Semi-structured interviews were held to increase our understanding of the survey outcomes and provide explanations. In addition, the interviews were used to identify barriers and facilitators of interprofessional collaboration in IPUs. Participants were asked how they would describe the composition of their IPU and why they considered other professionals to be part of the IPU or not. Other questions included “How do you collaborate with other professional groups in the IPU?” and “How do you communicate with other professional groups in the IPU?”. Interviews lasted between 30–60 minutes.

### 2.4. Data analysis

Questionnaires with missing responses on team perception, team diversity or one of the RC items were excluded. We calculated a relative team size deviation from the formal team size by dividing the perceived team size provided by each respondent by the formal team size, provided by the project managers responsible for the VBHC implementation. Furthermore, participants indicated which of the four professional groups they consider part of the team. A team diversity score was then calculated for each respondent by dividing the number of other professional groups that were considered part of the team by the maximum possible number of other professional groups. This resulted in scores ranging between 0 and 1, where 0 is a fully homogeneous team and 1 a fully heterogeneous team.

From the RC survey, overall RC scores were calculated by taking the average of the seven RC elements. RC scores were constructed for each dimension (communication and relationships) as the average of the respective item scores for each respondent, and ranged from 1.00 to 5.00. Both overall and dimension-specific scores were used to descriptively analyse the perceived degree of RC with the other professional groups [23,25,26]. Scores are presented for each professional group. Data were analysed using IBM SPSS 25.0.

Interviews were audiotaped and transcribed. A theoretical thematic analysis of a subset of the transcripts was performed by three researchers (DS, EA, PB) to identify patterns in the data using ATLAS.ti [27,28]. Codes were given to text segments in the transcripts, which were discussed among three authors (DS, EA, PB). After consensus was reached on the codes, these were grouped into subthemes and overarching themes. The extracted themes were discussed by the same three researchers (DS, EA, PB) until agreement was reached (see Additional File 1).

## 3. Results

Table 1 presents the descriptive characteristics of the participants. In total, 86 employees completed the survey (37%) of which the majority were female (63%). Most were medical specialist (59%) or nurses (23%). Cronbach’s alpha coefficients indicated good reliability for the overall RC scale (0.85) and its dimensions: communication (0.83) and relationships (0.84). Eighteen professionals participated in the semi-structured interviews: 7 medical specialists, 6 nurses, 2 administrative employees and 3 allied health professionals. After 18 interviews, no new perspectives and explanations were found, suggesting that the data were saturated. The results of the quantitative and qualitative studies are presented thematically below.

**Table 1:** Characteristics of survey and interview participants (n = 86; n = 18).


	N SURVEY (%)	N INTERVIEW (%)

Gender		

– Male	32 (37%)	5 (28%)

– Female	54 (63%)	13 (72%)

Profession		

– Medical specialist	51 (59%)	7 (39%)

– Nurse	20 (23%)	6 (33%)

– Administrative employee	6 (7%)	3 (17%)

– Allied health professional	9 (11%)	2 (11%)

Education		

– Senior general secondary education	3 (4%)	

– Secondary vocational education	3 (4%)	2 (11%)

– Higher professional education	23 (27%)	7 (39%)

– University education (master degree)	17 (20%)	1 (6%)

– University education (doctoral degree)	40 (47%)	8 (44%)


### 3.1. Team perception

**Figure 1:**
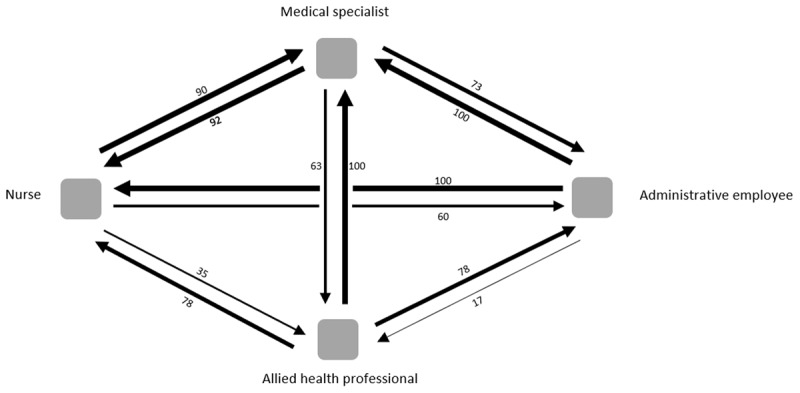
Visualisation of team perceptions among professional groups in IPUs. The arrows and numbers show the percentage of participants that considered other professional groups to be part of the IPU. For instance, 90% of the nurses considered a medical specialist to be part of their IPU.

Table 2 presents the average IPU size as perceived by each profession. Nurses indicated the largest IPU size (19.3 on average) and allied health professionals reported the smallest (13.2). The median relative deviation from the formal team size was effectively identical for three of the professional groups (1.13) and slightly lower for the administrative employees (1.06). As such, the professionals’ perceptions of IPU sizes were larger than on paper. The mean overall team diversity score was .79 (SD .22), with nurses perceiving the lowest diversity (.58) and allied health professionals the highest (.85). Figure 1 indicates the percentage of each professional group that considers each of the other professions to be part of their team. The quantitative results indicate that there are differences between professional groups regarding who they consider to be part of the IPU.

**Table 2:** Team perception in IPUs.


TOTAL RESPONDENTS GROUP (N = 86)		PERCEIVED TEAM SIZE				MEDIAN RELATIVE DEVIATION FROM FORMAL SIZE	TEAM DIVERSITY
	
		MEAN (SD)	MIN.	MAX.	MEDIAN		MEAN (SD)

1	Medical specialist (n = 51)	18.86 (10.51)	5	50	17	1.13	.75 (.31)

2	Nurse (n = 20)	19.25 (19.76)	4	95	15	1.13	.58 (.30)

3	Administrative employee (n = 6)	17.33 (6.86)	12	30	15	1.06	.72 (.14)

4	Allied health professional (n = 9)	13.22 (6.22)	4	20	15	1.13	.85 (.18)


The interviews revealed three factors that influence the consideration of who is part of the IPU: the degree of visibility, shared interest and involvement in the treatment of the patient.

First, seeing professionals from other groups at IPU meetings or while providing care contributes to perceiving them as part of the team, and vice versa:

“I only see her once a year or so, so she feels less like part of the team.” (MS)

“And well I see them, because we have a multidisciplinary team meeting every week so we are all together.” (MS)

“Despite the fact that 7 times out of 10 he is not there at patient meetings, I do see him as part of the team because he does work at our outpatient clinic together with the other ENT doctors on the team.” (N)

Second, professionals are inclined to consider others as part of the team when they experience a strong shared understanding of, or interest in, the care delivered by the IPU. This can contribute to a feeling of shared responsibility for the treatment of the patient.

“So I really consider this a team because it is partly the knowledge, but partly also the people’s real interest in the specific disease”. (MS)

“Those are the right people in the right seat. If I then speak to someone who does not understand at all what I am talking about, yes, then I find it very pointless. However, because everyone involved in this group of patients has the same approach, if you say something, it is also very logical.” (N)

A third factor that was reported to influence the perception of who is part of the IPU is the degree of involvement in the treatment of the patient. For medical specialists, professionals who are only partly involved are not considered part of the IPU. For instance, allied health professionals are often not considered to be part of the IPU by medical specialists because they are not involved in discussing the treatment plan for the patient or because they are not specifically appointed to one team.

“Except that I know what she looks like and that she is present, she is not involved. I send people to her just like I send someone to a dietician, but she doesn’t think along with us about the whole care process.” (MS)

“There is kind of a second circle […] they are not specific to our team. They are also available for other teams.” (MS)

In contrast, the other professional groups reported that who is involved, and therefore considered part of the IPU, depends less on the profession or involvement in the treatment planning, and more on the specific problems of a patient. They pictured a more flexible team in which professional groups are part of the IPU when they are involved in a specific stage of the treatment. Thus, for instance, professional groups could be seen as required and thereby part of the IPU in the first and third phases of a treatment, but not in the second phase.

“And then, as a result of the results of those PROMs or whatever the doctor hears in the waiting room, those other disciplines become involved. So, there is no standard package […] – as a result of what comes out of those conversations, we bring in other disciplines.” (N)

“Well I don’t think we have something to discuss with them every month, but indeed if something comes up we ask them or if they come up with ideas they will join, I don’t think it makes sense for them to always be there, but if they are needed for certain interesting activities, we’ll call them in.” (AE)

### 3.2. Relational Coordination

Table 3 displays the levels of RC among the professional groups. All share high degrees of overall RC (>3.81) with both medical specialists and nurses, whereas lower RC levels were reported by medical specialists and nurses when it came to perceptions of working with allied health professionals: medical specialists 2.95, nurses 2.96 and administrative employees 1.50. This pattern was also seen in both subdimension scores (relationship and communication), although the relationship scores were generally higher than the communication scores.

**Table 3:** Overall and dimension-specific RC scores.


WITH PROFESSION: IN EYES OF:	MEDICAL SPECIALIST	NURSE	ADMINISTRATIVE EMPLOYEE	ALLIED HEALTH PROFESSIONAL

Medical specialist				

– Overall RC	–	4.23	3.71	2.95

– Communication	–	4.12	3.45	2.76

– Relationships	–	4.38	4.05	3.20

Nurse				

– Overall RC	4.13	–	3.21	2.96

– Communication	3.96	–	3.10	2.81

– Relationships	4.35	–	3.37	3.17

Administrative employee				

– Overall RC	3.88	4.40	–	1.50

– Communication	3.63	4.33	–	1.54

– Relationships	4.22	4.50	–	1.44

Allied health professional				

– Overall RC	4.25	3.81	3.38	–

– Communication	4.14	3.64	3.19	–

– Relationships	4.41	4.04	3.63	–


These quantitative results indicate that the quality of interprofessional collaboration among IPU members is perceived differently among the professional groups. Insights from the interviews suggest that three factors might inhibit or facilitate high quality interaction among these professional groups in IPUs, which might in part explain the differences presented in Table 3: organisational structures, knowledge of each other’s expertise and way of communication.

First, the participants indicated that the quality of collaboration among IPU members is influenced by how well the hospital-wide organisational structures are equipped for working in an IPU. This has for example to do with access to each other’s schedules:

“We sometimes see that we send out PROM questionnaires for patients after the consultation took place. […] The patients should get it before the first consultation, but due to access restrictions, I can only afterwards see in the psychologist’s diary that they had an appointment. For the psychologist, this makes it impossible for them to optimally carry out the consultation.” (AE)

Other participants mentioned that organisational structures are often still set-up by department rather than IPU, which inhibits effective collaboration within the IPU:

“…you can only request a certain number of MRIs by department. Our vestibular schwannoma tumours need an MRI scan. Am I going to ask the ENT doctor to request this or the neurosurgeon? I think it is terrible that we are endlessly discussing who is going to request it […] So, give a tumour IPU a number of MRIs, so that we don’t have to start arguing with each other.” (MS)

Communication is more frequent between professional groups when they are located close to each other in the hospital, and collaboration thereby improved. A physical distance between professional groups hinders interaction and effective communication:

“Well, that I have to find where she is […] The idea is, if it’s a typical medical question that I just walk in and I ask “what did you just discuss?”. If that nurse is located elsewhere in the building, it is of course not very convenient from an organisational perspective.” (MS)

“In the sense that you run the risk of less easily running into each other when you want to, or that you have to find each other, which makes things a bit more difficult. And is that poor care? No, but you have a greater chance of delays during outpatient visits because you are further apart, and also because you need more time.” (MS)

“In the end, I just think it’s best if you all do the outpatient clinic in the same place, like we do. You know, sometimes the doctor is having an outpatient clinic and knocks on my door and says: “would you like to come with me for a moment because I’m now with Mrs A and she is really not well and we have to do something.” (AHP)

Second, having knowledge of each other’s expertise was reported by all the professional groups, but perceptions of how this affects interprofessional collaboration differed between the groups.

Nurses and allied health professionals feel that knowing each other’s roles is important in valuing each other’s activities and increasing the feeling of being heard and involved. They advocate for interactions in which they have the opportunity to think along in the process and experience a mutual interest in each other’s contributions:

“I think that the roles of nurses and doctors are too far apart. They have no idea what I do here all day. Not one has followed me for a day.” (N)

“Well, for example with the endocrinologist and me, when she has a consultation and there is a patient who just doesn’t feel well at all and then the doctor may say: do you have a minute? And then I join and say to the patient: I’m going to take over this part, you come with me for a moment and then you finish the conversation with the doctor.” (N)

“This morning a nurse sent me an email: I’m going to call this lady, can I talk to you for a moment? Yes, then I just think it’s really nice that she does that, you know. That she thinks, yes, I’m going to call that lady later, I see that she is being treated by that professional, before I call that lady, let’s coordinate. You know, she can read my report in HIX, but apparently she likes to tune in for a little while.” (AHP)

Medical specialists offered a more functional reason for the importance of knowing the expertise of other professional groups, namely for situations where they cannot solve the problem alone:

“Well, if you can’t figure it out yourself and you notice that the others can contribute to a solution from their own expertise, that is really nice. I don’t have all the expertise they have.” (MS)

Also, medical specialists reported that collaboration is, in general, not in their nature, which could also influence the quality of interprofessional collaboration:

“Well, because in general we are smart people who work very independently […] we are used to working one-to-one with the patient in the consultation room, not so much in a team.” (MS)

Third, another overarching theme, the ways of communication between professionals, was reported as having an influence on the quality of collaboration. Medical specialists, nurses and administrative employees all reported that face-to-face IPU meetings facilitate communication and thereby positively influence collaboration among team members. Team meetings in which professional groups are regularly present improve decision-making among professional groups and also ensure that professionals feel closer to each other and communicate more easily:

“Sometimes it is a puzzle because everyone has a very busy schedule and not everyone can be there, but when you are there you notice that decision-making is much easier and many points are discussed.” (AE)

In particular, nurses feel that medical specialists and allied health professionals are not always able to participate in IPU meetings and that this negatively impacts their communication:

“The difficult thing about it is that every now and then, because of course they [medical specialists] also have so many other things, communication is a bit difficult, for example because one person has been to the multidisciplinary team meeting and another has not.” (N)

In terms of how communication should take place, nurses prefer to communicate face-to-face with medical specialists because they find emails inconvenient:

It’s actually not convenient to send an email to medical specialists because you sometimes get overlooked in their mailbox and don’t get an answer. That’s annoying.” (N)

Further, both medical specialists and nurses indicated that communication should take place both formally and informally among IPU members. Nurses reported the importance of unplanned work-unrelated talks among IPU members. They would value more personal contact, for example by regularly showing interest by asking about one’s weekend, holidays or children. According to them, informal communication currently takes place mainly between nurses or between medical specialists. Medical specialists also acknowledged this importance, and feel they could improve on this point:

“We used to drink coffee together. It might sounds strange, but those are the moments you bond and come closer. Just talking about the holidays or ‘yes, it was busy over the weekend’, ‘yes, I was on duty it was indeed a busy shift’” (N)

“I have good contact with almost everyone in the IPU, but not so much on the personal level, I do not put enough time into that.” (MS)

## 4. Discussion

Providing the full cycle of care for a specific medical condition requires interaction between employees from diverse professional backgrounds [5]. The findings in this study showed that the perceptions of IPU sizes by those involved are larger than they actually are, and that the composition of IPUs and the quality of collaboration within these teams are perceived differently by the professional groups working in them.

Differences regarding IPU composition seemed to originate in perceptions of the roles of visibility, shared interest, and involvement. Participants were in agreement that one reason for considering someone to be part of an IPU or not, was the frequency at which they were seen. However, since acceptable frequency for someone to be considered part of the IPU was not specified, and might be interpreted differently, this might explain the differences in team perception among IPU members. Another commonly mentioned reason for considering someone as part of the team was the feeling of sharing interest for the IPU’s patient group. This ties in with the literature on team-based healthcare where it is argued that individuals with a common goal or collective interest are more inclined to consider each other to be part of the team and act as team members [29]. Finally, the level of involvement influences whether someone is considered part of the IPU or not, but this differed among the professional groups. Nurses and allied health professionals picture a relatively flexible team composition where incidentally involved professionals become part of the IPU when they are needed in the treatment process. However, medical specialists and administrative employees do not consider such professionals to be part of the IPU. As in earlier research in other domains, the medical specialists in this study seem to imagine a somewhat multi-layered team composition where some professional groups are part of the core team while others are on the periphery [15]. For instance, professionals will not be considered part of the core team if they do not work exclusively for that specific IPU. Previous research has concluded that the presence of these differing perceptions is likely to increase confusion among team members and potentially lead to lower performance [30]. Additionally, it is suggested that if two individuals consider each other to be part of the same team collaboration will be easier. On the basis of the above, we advocate for increased attention to be given to creating a shared perception of the IPU composition among its members.

We found that the quality of relationships and associated communication was generally considered highest by the medical specialists, followed by nurses, and poorest by the allied health professionals. This is again in line with earlier research in other domains than VBHC, where it is argued that high RC scores are likely from medical specialists and nurses, since these are considered the main coordinators of integrated care [18,31]. Our study identified three factors that influence the quality of collaboration in IPUs: organisational conditions, knowledge of each other’s expertise, and ways of communication.

The existing organisational structures were seen as limiting the ability of IPU members to collaborate effectively. For instance, the heads of clinical departments were accountable for decisions affecting the IPU in which their departments were involved, thereby limiting the mandate of IPU leaders and hindering the decision-making process and causing confusion regarding responsibilities. This finding is consistent with earlier research on challenges in VBHC implementation and re-emphasises the need for central responsibility within IPUs [3]. In addition, having IPU members working in different locations reduces the ability to collaborate effectively. Porter and Lee have previously urged IPU members to be co-located since this would facilitate the necessary communication and collaboration [5]. In line with the literature on co-location in primary healthcare settings [32,33], our study suggests that co-locating of IPU members would increase the ability to make effective use of time and resources. Based on these findings, we would encourage the embedding of IPUs in the administrative structure of the hospital and the centralisation of accountability within the IPUs themselves. Furthermore, we recommend facilitating shared work places for IPU members.

Knowing one another’s expertise is necessary for effective collaboration among IPU members, but why this is considered important, and what that knowledge is used for, seems to vary by profession. Nurses and allied health professionals needed to feel involved and want their work to be appreciated. This could be because these professional groups want to feel acknowledged, or because they feel that others have only a moderate understanding of their profession and therefore struggle with how to collaborate with them [34]. Medical specialists, on the other hand, view knowing each other’s expertise as vital in deciding who to include, or leave out, in the treatment process [16]. As such, nurses and allied health professionals seem to have a somewhat more personal view of the importance of knowledge of other’s expertise whereas medical specialists have a rather functional and practical view. This might be due to the hierarchical system that still exists where nurses seek approval and appreciation since they feel second rate compared to medical specialists [35,36,37,38,39]. Further, medical specialists often see themselves as highly responsible for patient treatment and therefore feel they should decide who to involve in a patient’s treatment, which could explain their functional view on the importance of knowing each other’s expertise [40]. Based on the above, we would emphasise the value in learning about each other’s expertise and role in the IPU, and about the differing reasons as to why this is seen as important with respect to the IPU. This could be achieved, for instance, through training programs, actively sharing such information among IPU members or through observing each other’s work activities.

Finally, frequent formal and informal communication was seen as essential for good quality collaboration in IPUs, and that this needed to be improved. IPU meetings were viewed as facilitators of formal communication, and whether or not members were present at IPU meetings was found to greatly impact the quality of interprofessional collaboration and decision-making among IPU members. This is especially effective for team performance if all the professional groups are represented [41]. Frequent informal communication is also seen as important, but this currently happens mainly within the individual professional groups. Earlier research has suggested that this is due to groups of nurses and medical specialists forming cliques with their own norms, values and cultures, and thereby insulating themselves from other professional groups [39]. This might explain the differences in perceived communication between the diverse professional groups, identified in Table 3, for instance, the perceived communication between medical specialists and allied health professionals. As informal communication is known to be valuable for collaborative practice [42], and communication in IPUs should especially cross professional boundaries [43], we would advise identifying and addressing factors, such as the spatial organisation of wards [42], that can impede informal communication.

The results of this study highlight that high-quality interprofessional collaboration between different professional groups in IPUs cannot be assumed. Based on the findings, we encourage managers to actively facilitate interprofessional collaboration among IPU members. This can be done, for instance, by creating a clear division of tasks and responsibilities between IPU members, facilitating shared workspaces for IPU members, and stimulating informal team activities for IPU members.

At the policy level, we would advise policymakers to promote interprofessional education, as this is expected to enhance shared knowledge and thereby the quality of interprofessional collaboration between professionals working in multidisciplinary teams [44].

### 4.1. Limitations

Certain limitations should not be overlooked when considering our findings. First, both the survey and interview samples consisted mainly of medical specialists and nurses. This might have affected the results and the conclusions that were drawn from this study. However, the population of professionals that was relevant for the study consisted mostly of medical specialists and nurses, with relatively few administrative employees and allied health professionals. In part because of these concerns, the results are presented by professional group.

Second, the small sample size and limited number of individuals per team did not allow for a comparison within or between teams, limiting the generalizability of our findings. Despite efforts to achieve a large sample size, this remained small, partly because eligible participants didn’t complete the survey either because they didn’t consider themselves part of the IPU, or felt they did not collaborate enough with other professional groups. Future research could investigate team perception and collaboration among professional groups within a specific IPU. Further, since the results in this study suggest that the quality of interprofessional collaboration depends on coordination of knowledge and communication on a team-level, future research could extent this by considering how other team-level characteristics, such as leadership or role division, influence interprofessional collaboration in IPUs.

Third, the results relied on self-reported data, which may be subject to social desirability bias or recall bias. This may have affected the results by over-reporting socially desirable behaviours or under-reporting undesirable attitudes. However, the surveys were anonymous and self-administered, reducing the likelihood of desirable answers. Also, the researcher conducting the interviews was not familiar with the participants and ensured their anonymity in the study, which allowed the participants to answer truthfully.

Fourth, we have only carried out descriptive analyses of the quantitative data and have not explored associations. Nevertheless, the qualitative results of this study suggest a possible relationship between team perception and relational coordination. For example, professional groups that do not consider each other to be part of the IPU perceive similarly low levels of RC, whereas professional groups that perceive each other as part of the IPU have high levels of RC in common. This suggests a positive correlation between team perception and RC, and future research could investigate to what extent a relationship exists between team perception and RC in IPUs. Since we used a cross-sectional design, we were unable to observe how interprofessional collaboration in IPUs and the suggested causal relationships between variables evolve. Future research with a longitudinal design is needed to better understand this aspect of interprofessional collaboration within the IPU.

## 5. Conclusions

Organising care around medical conditions in IPUs, where professionals from different backgrounds are expected to collaborate effectively, is challenging given that shared perceptions of team composition and of high quality interprofessional collaboration are not evident. In practice, professionals’ understanding of the IPU composition and the collaboration among IPU members influences their interpretations and actions. The differing perceptions are likely to result in differences in the expectations of IPU members and their associated collaborative actions. Consequently, we emphasise the need for training programmes for IPU members in which the focus is on learning about and clarifying the expertise of the various IPU members and their role in the IPU, since this will likely improve interprofessional collaboration among IPU members. To enhance the impact of these training programmes they should be expanded with discussions between IPU members on collaboration preferences and needs. Finally, more research on the collaborative practices of IPU members would provide further guidance for IPU members and managers responsible for VBHC implementation on how to achieve effective VBHC delivery within an IPU environment.

## Data Accessibility Statement

The datasets generated during and/or analyzed during the current study are not publicly available, but are available from the corresponding author on reasonable request.

## Additional File

The additional file for this article can be found as follows:

10.5334/ijic.7516.s1 Additional file 1. Thematic analysis.
