# Supplementary material for: Differing Professional Perspectives on the Interprofessional Collaboration in IPUs: A Mixed-methods Study
Source: Int J Integr Care. 2023 Aug 10;23(3):5. doi: 10.5334/ijic.7516 (PMC10418149; doi:10.5334/ijic.7516)
Supplement: Additional file 1. — Thematic analysis. [file ijic-23-3-7516-s1.pdf]

Additional file 1

**Thematic analysis**		
**Code**	**Sub-theme**	**Theme**
Involved in each stage of the treatment	Involvement	Team perception
Asking to join in the treatment		
Directly involved in patient care		
Present at team meetings	Visibility	
Present at the outpatient clinic		
Shared interest in medical condition	Shared interest	
Know each other's work activities	Knowledge of each other's expertise	Interprofessional collaboration
Put in effort for each other's work		
Feeling involved in the treatment process		
Proving your knowledge and skills		
Differences in background and education		
Getting to know each other's work activities over time		
Present at multidisciplinary team meetings	Degree of communication	
Level of informal contact		
Feeling of psychological safety		
Access to each other's diaries	Organisational structures	
Physical distance		
Department focused structures		
